# Diversity in rest–activity patterns among Lake Malawi cichlid fishes suggests a novel axis of habitat partitioning

**DOI:** 10.1242/jeb.242186

**Published:** 2021-04-15

**Authors:** Evan Lloyd, Brian Chhouk, Andrew J. Conith, Alex C. Keene, R. Craig Albertson

**Affiliations:** 1 Department of Biological Science, Florida Atlantic University, Jupiter, FL 33401, USA; 2 Department of Biology, University of Massachusetts, Amherst, MA 01003, USA

**Keywords:** Cichlid, Circadian rhythm, Comparative behavior

## Abstract

Animals display remarkable diversity in rest and activity patterns that are regulated by endogenous foraging strategies, social behaviors and predator avoidance. Alteration in the circadian timing of activity or the duration of rest–wake cycles provide a central mechanism for animals to exploit novel niches. The diversity of the >3000 cichlid species throughout the world provides a unique opportunity to examine variation in locomotor activity and rest. Lake Malawi alone is home to over 500 species of cichlids that display divergent behaviors and inhabit well-defined niches throughout the lake. These species are presumed to be diurnal, though this has never been tested systematically. Here, we measured locomotor activity across the circadian cycle in 11 Lake Malawi cichlid species. We documented surprising variability in the circadian time of locomotor activity and the duration of rest. In particular, we identified a single species, *Tropheops* sp. ‘red cheek’, that is nocturnal. Nocturnal behavior was maintained when fish were provided shelter, but not under constant darkness, suggesting that it results from acute response to light rather than an endogenous circadian rhythm. Finally, we showed that nocturnality is associated with increased eye size after correcting for evolutionary history, suggesting a link between visual processing and nighttime activity. Together, these findings identify diversity of locomotor behavior in Lake Malawi cichlids and provide a system for investigating the molecular and neural basis underlying variation in nocturnal activity.

## Abstract (precis)

**Summary:** Cichlids show a remarkable diversity in morphology and behavior. Cichlid species exhibit differences in strength and polarity of activity rhythms, revealing a new axis of habitat partitioning.

## INTRODUCTION

Animals display remarkable diversity in rest and activity patterns. The timing of locomotor activity and rest can differ dramatically between closely related species, or even between individuals of the same species, raising the possibility that it can be adaptive and subject to selection (Brown et al., 2018; Duboué et al., 2011; Hammond et al., 2018). Indeed, circadian regulation of locomotor activity is strongly associated with foraging strategies, social behaviors and predator avoidance, which are critical factors in organismal fitness (Siegel, 2005; Vaze and Sharma, 2013). Alteration in the circadian timing of activity or the duration of rest–wake cycles provide a central mechanism for animals to exploit novel niches. Indeed, there is some evidence that alterations in daily rhythms can be a major factor in speciation events. For example, in two closely related species of the tephritid fruit fly, hybridization in the wild appears to be prevented only by circadian differences in mating times (Raphael et al., 2019; Smith, 1979).

Across phyla the timing of rest and activity is regulated by a circadian clock that persists under constant conditions, as well as acute response to environmental cues that include light and food availability (Huang et al., 2011). For example, many teleost species display robust diurnal locomotor rhythms including the goldfish (*Carassius auratus*), the Mexican tetra (*Astyanax mexicanus*) and the zebrafish (*Danio rerio*) (Duboué et al., 2011; Iigo and Tabata, 1996; Zhdanova et al., 2001). Conversely, limited examples of nocturnal teleosts have been identified including the plainfin midshipman (*Porichthys notatus*), the Senegalese sole (*Solea senegalensis*) and the doctor fish (*Tinca tinca*) (Bayarri et al., 2004; Feng and Bass, 2016; Oliveira et al., 2009). Further, other species such as the Mexican cavefish (*A. mexicanus*) and the Somalian cavefish (*Phreatichthys andruzzii*) have largely lost circadian regulation of behavior (Cavallari et al., 2011; Beale et al., 2013). Despite these conspicuous differences, variation in rest and activity patterns have not been well described within a lineage that inhabits a shared environment. Moreover, the ecological basis of such variation and its relationship to niche exploitation have not been studied systematically.

Cichlids represent a leading model for investigating the evolution of development, morphology and complex behavior. In Lake Malawi alone, there are many hundreds of cichlid species, inhabiting a diversity of environmental and feeding niches (Turner et al., 2001). Cichlid species exhibit a high degree of habitat fidelity and partition their environment along discrete ecological axes, including distinct biotic (food availability, predation and parasites) and abiotic (light, water chemistry) environments that play a critical role in the origins and maintenance of cichlid biodiversity (Albertson, 2008; Huber et al., 1997; Karvonen et al., 2018; Malinsky et al., 2015; Parnell and Todd Streelman, 2011; Terai et al., 2017). Lake Malawi is home to many cichlid predators, which are hypothesized to influence the behavior, distribution and diversification of cichlid species in the lake (Fryer, 1959). For example, the Cornish jack, *Mormyrops anguilloides*, is a large nocturnal predator that hunts cichlids in the intermediate and near-shore rocky habitat. *Mormyrops anguilloides* are weakly electric fish that hunt at night using electrical pulses thought to be undetectable by cichlids (Arnegard and Carlson, 2005). Field studies on this predatory behavior have suggested that near-shore cichlids are largely diurnal (Arnegard and Carlson, 2005), in agreement with the notion that rest represents a form of adaptive inactivity that allows for predator avoidance (Siegel, 2009). Deviations from diurnal activity have been noted for New World cichlids, which exhibit nocturnal parental care of eggs (Reebs and Colgan, 1991, 1992), and the ability of some Malawi cichlids to forage in low-light conditions, via widened lateral line canals, suggests the potential for nocturnal behaviors to evolve in this group (Schwalbe et al., 2012; Edgley and Genner, 2019). Given that Malawi cichlids exhibit an impressive magnitude of diversity in an array of anatomical and behavioral traits, we predicted that they may also exhibit high magnitudes and continuous variation in rest–activity patterns. Indeed, this could represent an important, but underappreciated, dimension of habitat partitioning.

The development of automated tracking of locomotor activity in fish species has been applied for the study of sleep and locomotor activity in zebrafish and Mexican cavefish (Jaggard et al., 2019). These methodologies provide the opportunity for comparative approaches that examine differences in activity between populations, and across contexts. Here, we extended this methodology to study sleep across 11 species of cichlids, from diverse habitats. Our choice of species focused on the near-shore rock-dwelling clade of Malawi cichlids (i.e. *mbuna*), but we also included representative species from other lineages within the lake. Our goal was not to characterize the evolution of rest–activity patterns per se, but rather to better understand the degree and type of variation exhibited by this group. We identified robust variation in the quantity, as well as the circadian timing, of rest and activity. In addition, this analysis reveals, for the first time, a nocturnal species of Malawi cichlid, suggesting that circadian regulation of activity may provide a mechanism for niche exploitation in African cichlids. In support of this assertion, we demonstrated further that activity levels are associated with an eco-morphological and behavioral trait. Together, these findings suggest that cichlids can be used as a model to study the evolution of, and molecular mechanisms for, variation in locomotor rhythms.

## MATERIALS AND METHODS

### Fish stocks and husbandry

Cichlids used for experiments were reared following standard protocols approved by the University of Massachusetts Institutional Animal Care and Use Committee. Cichlids were housed in the Albertson fish facilities at the University of Massachusetts, Amherst at a water temperature of 28.5°C, kept on a 14 h:10 h light:dark cycle, and fed a diet of a flake mixture consisting of ∼75% spirulina algae flake and ∼25% yolk flake twice a day. Cichlids were derived from wild-caught animals that were either reared at the Albertson fish facilities [*Labeotropheus trewavasae* (F_2_ generations from wild), *Maylandia zebra* (F_3_) and *Tropheops* sp. ‘red cheek’ (F_2_)] or obtained through the aquarium trade (*Sciaenochromis fryeri*, *Copadichromis trewavasae*, *Aulonocara stuartgranti*, *Dimidiochromis compressiceps*, *Labeotropheus fuelleborni*, *Iodotropheus sprengerae*, *Tropheops* sp. ‘red fin’ and *Tropheops* sp. ‘elongatus Boadzulu’)*.* We have used cichlids for genetic and developmental experiments up to the F_5_ generation, and have not noticed any deleterious effects of inbreeding (e.g. Conith et al., 2020b). The same set (or subset) of animals was used for all analyses. Because of the nature of the testing tanks (see below), all fish were tested at the late juvenile stage, making sex determination difficult to assess at the time; however, after the experiments took place, stocks were grown out and it could be confirmed that sex ratios were 50:50 on average.

Recent genomic analyses have shown that the Lake Malawi cichlid species flock is composed of three distinct radiations: (1) *mbuna*, (2) shallow benthic, deep benthic and *utaka*, and (3) pelagic (Malinsky et al., 2018). Phylogenetic signal within each radiation is confounded by the incomplete sorting of ancestral alleles and ongoing gene flow between species (Brawand et al., 2014; Malinsky et al., 2018). Here, we focus on the *mbuna* radiation, but also include four species from the shallow benthic, deep benthic and *utaka* radiation (i.e. *S. fryeri*, *C. trewavasae*, *A. stuartgranti* and *D. compressiceps*), which we refer to as ‘non-*mbuna*’ for simplicity.

### Behavioral analysis

Twenty-four hours prior to the beginning of each experiment, juvenile fish were transferred from their home tanks into 10 liter tanks (Carolina Biologicals) with custom-designed partitions that allowed for up to three fish to be individually housed in each tank. After 24 h of acclimation, fish were fed, tanks were given a 50% water change to maintain water quality, and behavior was recorded for a 24 h period beginning at zeitgeber time (ZT) 3, 3 h after light onset. Videos were recorded at 15 frames s^−1^ using a USB webcam (LifeCam Studio 1080p HD Webcam, Microsoft) through the video processing software VirtualDub (v1.10.4). To allow for recording during the dark period and provide consistent lighting throughout the day, cameras were modified by removing their infrared filters and replacing with IR long-pass filters (Edmund Optics Worldwide), and tanks were illuminated from behind using infrared light strips (Infrared 850 nm 5050 LED Strip Light, Environmental Lights). All behavioral and video recording procedures were based on those developed for use in measuring activity and sleep in *A.* *mexicanus* (Jaggard et al., 2019).

For experiments testing the effect of shelter on locomotor activity, a small PVC tube (3×1 inch, length×outer diameter) was added to each chamber at the beginning of the acclimation period. For experiments testing the effect of light, fish were acclimated to their tanks on a normal 14 h:10 h light:dark cycle, and then recorded in 24 h of darkness. Following acquisition, recordings were processed in Ethovision XT 15 (Noldus) to extract positional data for individual fish throughout the 24 h period, and these data were used to calculate velocity and locomotor activity, as previously described (Yoshizawa et al., 2015).

To identify variation in rest and activity patterns across cichlid species, positional data were exported from Ethovision and analyzed using a custom-made Perl script (v5.10.0) and Excel Macro (Microsoft). A threshold of 4 cm s^−1^ was set to correct for passive drift of the animal; any reading over this threshold was classified as active swimming and used to calculate velocity. Any period of inactivity lasting greater than 60 s was classified as a ‘rest’ bout, and the time and duration of each rest bout were recorded to generate profiles of rest throughout the day.

### Measurements of eye size

Fish were imaged using a digital camera (Olympus E520) mounted to a camera stand. All images included a ruler. Using the program ImageJ (Schneider et al., 2012), measures of standard length, head length and eye area were obtained for each fish. Eye size was measured in fish used in the behavioral analysis. In addition, when possible, we augmented these samples with wild-caught animals from the Albertson laboratory collections. In particular, we added wild-caught samples to the *L. fuelleborni*, *M. zebra*, *Tropheops* sp. ‘red cheek’ and *Tropheops* sp. ‘red fin’ populations.

### Phylogenetic comparative methods

Given the shared evolutionary histories of our cichlid taxa, we used phylogenetic regression to account for this non-independence (Freckleton et al., 2002). We used a time-calibrated phylogeny of cichlids that included all but two of our taxa to perform all comparative methods (McGee et al., 2020). There were two taxa absent from the tree that we had data for, *Tropheops* sp. ‘red cheek’ and *Tropheops* sp. ‘red fin’. The cichlid tree of McGee et al. (2020) revealed well-supported monophyly of *Tropheops*, with short branch lengths. Based on this evidence, and other previously published trees of *Tropheops* (i.e. Conith et al., 2020a,b), we substituted the trait values of *Tropheops* sp*.* ‘red cheek’ and *Tropheops* sp*.* ‘red fin’ for values of two taxa present in the tree, *T. gracilior* and *T. microstoma*. Once the tree was finalized (shown in Fig. S1), we used the R (https://www.r-project.org/) phylolm function in the phylolm package (v. 2.6.2) to perform all future phylogenetic regressions (Tung Ho and Ané, 2014). We used Pagel's λ to conduct all transformations of the phylogenetic covariation matrix (Pagel, 1999), and used maximum likelihood to find the best-fitting λ parameter estimate for our tree. The effect of a λ transformation is to reduce or eliminate phylogenetic signal in the data by multiplying each internal branch by the λ estimate found via maximum likelihood. The phylolm function can then incorporate the transformed covariance structure between taxa residuals into the calculation of slope and intercept estimates.

Given that relative eye size exhibits strong allometric effects (Howland et al., 2004), we first performed a square root transformation on the raw eye area values to linearize the data, and then executed a phylogenetic size correction between standard length and linearized eye area. Once we had obtained residual eye areas we performed two further phylogenetic regressions using the λ transformation, we first regressed residual eye area by total rest time, then regressed residual eye area by the activity change ratio.

Finally, we performed a phylogenetically corrected ANOVA (pANOVA) to assess whether the degree of territoriality exhibited by a taxon impacted the total distance traveled. Given the necessity of more territorial taxa to patrol continuously so to ward off conspecifics or potential threats, we predicted territorial taxa would cover more ground. We used the R function aov.phylo from the geiger package (v. 2.0.6.1) to perform the pANOVA (Harmon et al., 2008). The pANOVA assesses differences between territory grouping assignments by simulating total locomotor activity data over the cichlid tree under Brownian motion 1000 times, then comparing that simulated null distribution of test statistics with the empirical data to obtain significance.

### Statistics and analysis

One-way ANOVAs were carried out to identify inter-specific differences in overall locomotor activity, average waking velocity, rest duration and total time in shelter. Equality of variance between groups was determined using Levene's median test, and normality was assessed by calculating the residuals of the pooled data, and plotting on a quantile–quantile graph to visually assess normality. To identify differences between multiple conditions, such as activity in the light versus dark, or shelter versus no-shelter conditions, a two-way ANOVA was carried out, and followed by Šidák's multiple comparisons *post hoc* test. To identify significant rhythms in activity across the day–night cycle, an ‘activity change ratio’ (*A*_R_) was calculated as follows:(1)
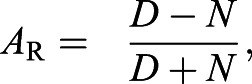
where *D* and *N* are average hourly activity during the day and night. This procedure is conceptually similar to the ‘diurnality index’ described by Hoogenboom et al. (1984), with +1 indicating total diurnality and −1 indicating total nocturnality, except that our calculations used direct measurements of activity instead of trapping frequency. To identify significant rhythmicity in activity, one-sample *t*-tests were performed. To identify differences between the *mbuna* and non-*mbuna* groups, nested ANOVAs were performed. Unless otherwise stated, all statistical analyses were carried out using InStat software (GraphPad Prism 8).

## RESULTS

### Variation in activity and rest behaviors

**Fig. 1.**
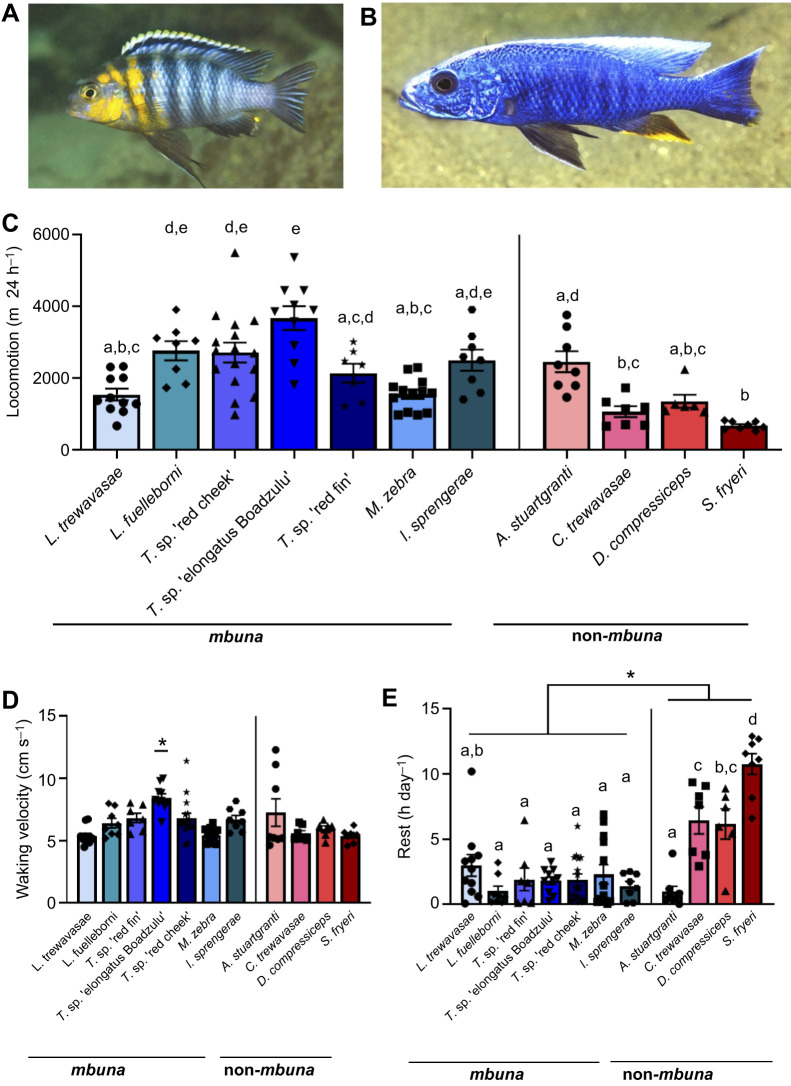
**Evolved differences in locomotor activity between cichlid species.** (A) Example of *Tropheops* sp. ‘red cheek’ of the *mbuna* clade. (B) Example of *Sciaenochromis* *fryeri* of the non-*mbuna* group. Images by Ad Konings, Cichlid Press. (C) Total locomotor activity over 24 h varies significantly across 11 cichlid species: *Aulonocara stuartgranti*, *Copadichromis trewavasae*, *Dimidiochromis compressiceps*, *Iodotropheus sprengerae*, *Labeotropheus fuelleborni*, *Labeotropheus trewavasae*, *Maylandia zebra*, *Sciaenochromis fryeri*, *Tropheops* sp. ‘red cheek’, *Tropheops* sp. ‘red fin’ and *Tropheops* sp. ‘elongatus Boadzulu’ (one-way ANOVA: *F*_10,91_=12.38, *P*<0.0001). *Mbuna* species trend towards higher activity than non-*mbuna* species, although this relationship does not reach significance (nested ANOVA, *F*_1,9_=4.469, *P*=0.064). (D) Waking velocity over 24 h is significantly elevated in only one species of cichlid, *Tropheops* sp. ‘elongatus Boadzulu’ (one-way ANOVA, *F*_10,89_=5.431, *P*<0.0001). (E) Consolidated periods of rest (>60 s) vary significantly across *mbuna* and non-*mbuna* groups (nested ANOVA, *F*_1,9_=7.883, *P*=0.0205). Error bars represent ±1 s.e.m. Data points in the bar graphs represent individual animals.

To measure variation in activity across Lake Malawi cichlids, we compared the locomotor activity in 11 different species, across eight genera. These species were selected for diversity in habitat, behavior and lineage representation. We sampled more deeply in the rock-frequenting *mbuna* clade (*n*=7 species, *n*=4 genera), which occupy a complex, three-dimensional habitat characterized by a high density of cichlid individuals (Fig. 1A). In addition, we analyzed activity patterns in four non-*mbuna* species, which occupy the intermediate to open-water habitat (Fig. 1B). Following an initial 24 h period of acclimation, activity was recorded in individually housed juvenile fish across 24 h in standard light:dark conditions, with infrared lighting used to monitor locomotor activity during the night as previously described in *A. mexicanus* (Yoshizawa et al., 2015). Quantification of total locomotor activity over 24 h identified marked variation across species, with certain species (i.e. *S. fryeri*) exhibiting lower activity than all other species tested, while the activity of others (i.e. *Tropheops* sp. ‘elongatus Boadzulu’) was significantly greater than most other species (Fig. 1C). Notably, variation in mean activity was continuously distributed between these two extremes. In addition, there was a division between *mbuna* and non-*mbuna* species, with *mbuna* species trending towards increased locomotion relative to non-*mbuna* species (*P*=0.0637).

To determine whether these differences were due to hyperactivity or differences in rest, we measured the average waking velocity for each population. Among all species tested, only one (*Tropheops* sp. ‘elongatus Boadzulu’) displayed significantly higher swimming velocity, suggesting the bulk of the variation among species is due to differences in rest/activity regulation (Fig. 1D). In agreement with this notion, there were significant inter-specific differences in the duration of rest bouts lasting greater than 1 min (Fig. 1E). The majority of species displayed very little rest, averaging less than 3 h day^−1^, while three species, *C. trewavasae*, *D. compressiceps* and *S. fryeri* (all non-*mbuna*), spent significantly longer resting than other species tested. The average rest duration of *S. fryeri* was up to 10-fold different from that of other species tested. Together, these findings suggest that differences in total locomotor activity between cichlid species are largely attributable to differences in rest. Notably, *mbuna* species together rested significantly less than non-*mbuna* species (Fig. 1E), possibly reflecting adaptation to the near-shore rocky habitat. Support for this possibility, as opposed to lineage-specific effects, is the observation that *A. stuartgranti*, a non-*mbuna* species that co-occurs with *mbuna*, rests less than other non-*mbuna* species (Fig. 1E).

### Variation in patterns and magnitudes of rhythmic activity

**Fig. 2.**
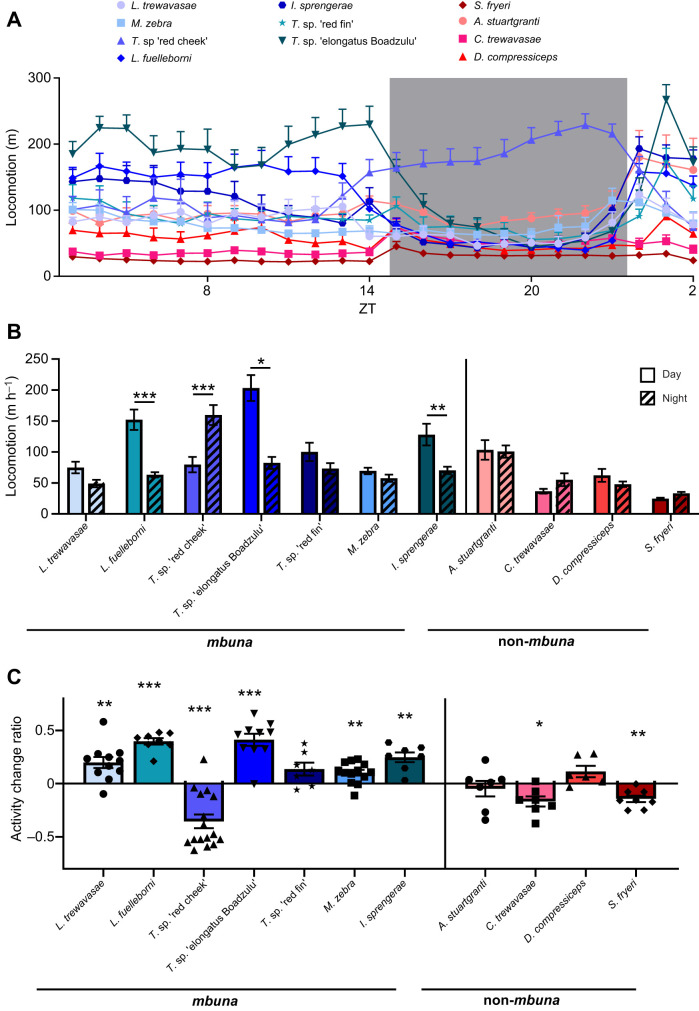
**Variation in daily activity rhythms across the day–night cycle.** (A) Activity profiles of all species tested across 24 h, beginning at ZT3. (B) Several species exhibit significantly increased activity during the subjective day (*L. fuelleborni*, *Tropheops* sp. ‘elongatus Boadzulu’, *I. sprengerae*) while a single species exhibits increased locomotor activity during the subjective night (*Tropheops* sp. ‘red cheek’) (two-way ANOVA, *F*_10,91_=19.56, *P*<0.0001). (C) Activity change scores, calculated as the difference between daytime and nighttime activity, divided by their sum, reveal differences in day/night preference across cichlid species [one-sample *t*-test; *L. trewavasae* (*t*_12_=3.839, *P*=0.0033), *L. fuelleborni* (*t*_7_=12.93, *P*<0.0001), *Tropheops* sp. ‘red cheek’ (*t*_15_=5.524, *P*<0.0001), *Tropheops* sp. ‘elongatus Boadzulu’ (*t*_9_=7.182, *P*<0.0001), *M. zebra*, (*t*_12_=3.790, *P*=0.0026), *I. sprengerae* (*t*_6_=5.374, *P*=0.0017), *C. trewavasae* (*t*_6_=3.555, *P*=0.012), *S. fryeri* (*t*_7_=4.693, *P*=0.0022)]. Error bars represent ±1 s.e.m. Data points represent individual animals.

To determine whether there are differences in circadian modulation of activity, we compared activity over the light:dark cycle (Fig. 2A). We found evidence for strong diurnal activity in three *mbuna* species (*L. fuelleborni*, *Tropheops* sp. ‘elongatus Boadzulu’ and *I. sprengerae*), while activity did not significantly differ based on light or dark phases in seven species tested (Fig. 2B). A single species, *Tropheops* sp. ‘red cheek’, was significantly more active in the night, providing the first evidence for nocturnality in a Lake Malawi cichlid (Fig. 2B). To account for variation in total locomotion between fish of different species, we quantified preference for light and dark activity for each individual tested. In agreement with quantification of average locomotor activity, *Tropheops* sp. ‘red cheek’ had significantly greater preference for nighttime activity, whereas *L. fuelleborni*, *M. zebra*, *Tropheops* sp. ‘elongatus Boadzulu’ and *I. sprengerae* had significantly greater preference for daytime activity (Fig. 2C). This analysis also suggests a preference for diurnal activity in *L. trewavasae*, and for nocturnal activity in two additional non-*mbuna* species (*C. trewavasae* and *S. fryeri*).

In other diurnal teleosts, such as *A. mexicanus* and *D. rerio*, rest is largely consolidated during nighttime (Duboué et al., 2011; Gandhi et al., 2015). To quantify time-of-day differences in rest across cichlid species, we compared the average amount of rest per hour across the 14 h:10 h light:dark periods (Fig. S2). This analysis is largely in agreement with analysis of locomotor activity, with day-active species consolidating rest during the dark period, and vice versa.

**Fig. 3.**
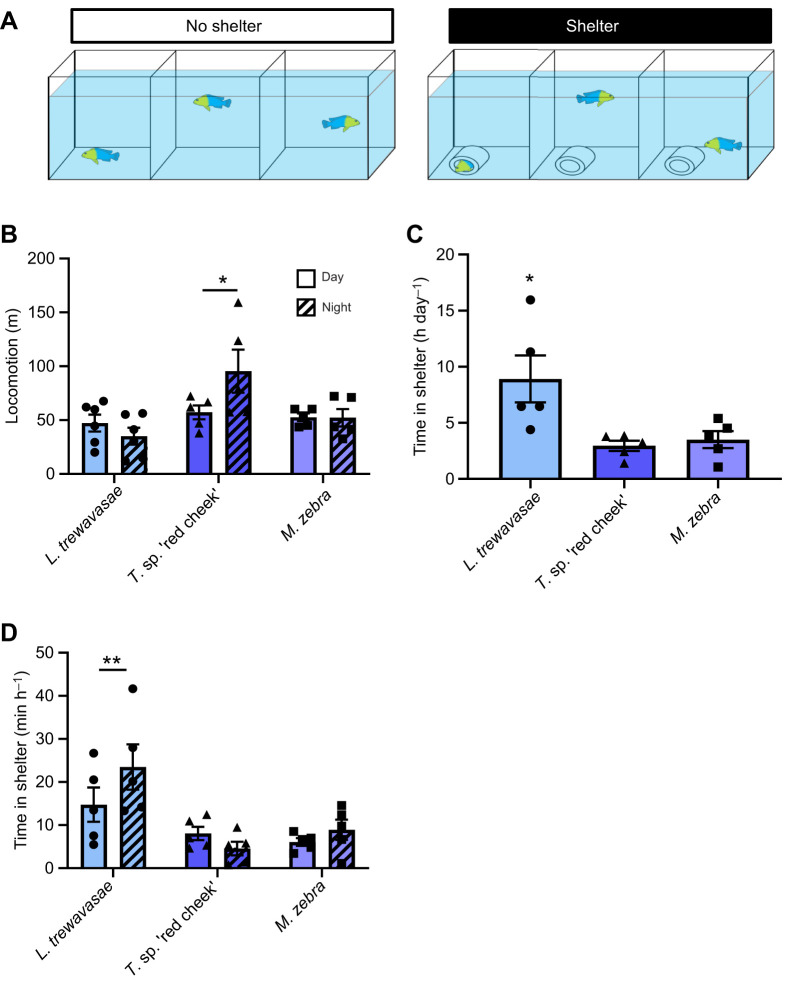
**Presence of shelter does not affect the nocturnal phenotype of *Tropheops* sp. ‘red cheek’.** (A) Compared to our previous analyses (‘no shelter’), select cichlid species were tested for locomotor activity over 24 h in the presence of a 3-inch PVC tube (‘shelter’), providing the option to take shelter at any point during the day. (B) *Tropheops* sp*.* ‘red cheek’ maintain a bias for nocturnal activity in the presence of shelter (two-way ANOVA, *F*_2,12_=7.9, *P*=0.0065). (C) *Labeotropheus* *trewavasae* exhibit significantly greater preference for the shelter relative to other species tested, consistent with knowledge of the species' ecological niche (one-way ANOVA, *F*_2,12_=6.305, *P*=0.0134). (D) Preference for shelter increases at night only in *L. trewavasae* (two-way ANOVA, *F*_2,12_=7.9, *P*=0.0065). Error bars represent ±1 s.e.m. Data points represent individual animals.

Because *Tropheops* sp. ‘red cheek’ is a highly territorial and aggressive species (Maruyama et al., 2010; Ribbink et al., 1983), it is possible that its nighttime activity represents a search strategy for locations that provide shelter, as opposed to a natural reflection of activity patterns. To differentiate between these possibilities, we provided each animal with a 3-inch cylindrical shelter (PVC piping), and measured behavior across light and dark conditions (Fig. 3A). We analyzed the total activity across the circadian cycle, as well as time spent in the shelter in *Tropheops* sp. ‘red cheek’, as well as in *L. trewavasae* and *M. zebra*, closely related *mbuna* species that co-occur with *Tropheops* sp. ‘red cheek’. These two species also exhibited lower and indistinguishable activity levels during the day and night, and we were interested to see whether the addition of shelter would alter this pattern*.* When provided a hiding spot, *Tropheops* sp. ‘red cheek’ remained robustly nocturnal, while *M. zebra* and *L. trewavasae* did not show light/dark preference, which is consistent with their activity patterns in the absence of shelter (Fig. 3B). We quantified the total time animals spent within the shelter and found that *L. trewavasae* spent significantly more time in the shelter than *M. zebra* and *Tropheops* sp. ‘red cheek’ (Fig. 3C), which is consistent with this species' behavior in the wild. *Labeotropheus* *trewavasae* has an elongated and dorso-ventrally compressed body plan, and exhibits habitat preference for cracks and crevices in the wild (Konings, 2001; Ribbink et al., 1983). Further, *L. trewavasae* spent more time in the shelter during the night period, consistent with an increased need to avoid nocturnal predators (Fig. 3D). Conversely, there were no differences in shelter preference between light or dark periods for *M. zebra* and *Tropheops* sp. ‘red cheek’. Together, these findings suggest that the presence of a shelter does not significantly affect the activity pattern of the cichlid species tested, and that the nocturnal locomotor activity of *Tropheops* sp. ‘red cheek’ does not represent a search for shelter.

**Fig. 4.**
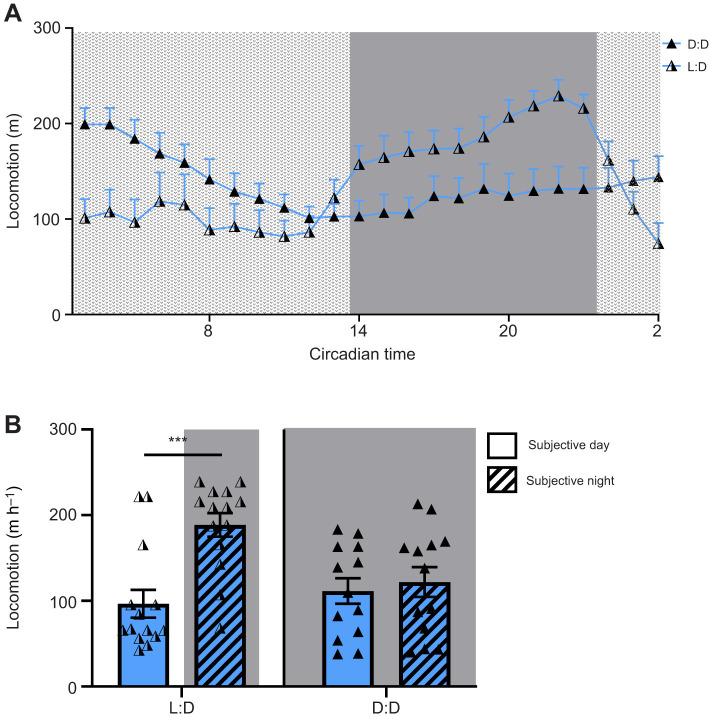
**Locomotor activity in *Tropheops* sp. ‘red cheek’ is modulated by the presence/absence of light.** (A) Activity profiles of *Tropheops* sp. ‘red cheek’ under a 14 h:10 h light:dark cycle (half-shaded triangles) and in constant darkness (fully shaded triangles). (B) Under a 14 h:10 h light:dark cycle, *Tropheops* sp. ‘red cheek’ increase activity during the day; in 24 h of darkness, activity remains consistent throughout the 24-h period (two-way ANOVA, *F*_1,22_=13.68, *P*=0.013). Error bars represent ±1 s.e.m. Data points represent individual animals.

It is possible that the nocturnal locomotor behavior of *Tropheops* sp. ‘red cheek’ is due to an endogenous circadian rhythm or a differential response to light. To distinguish between these possibilities, we measured locomotor activity under constant dark conditions. Briefly, fish were acclimated under standard 14 h:10 h light:dark conditions, then activity was recorded for 24 h under constant darkness (Fig. 4A). While *Tropheops* sp. ‘red cheek’ significantly increase their activity during the dark period under light:dark conditions, there was no difference between light and dark activity under constant darkness (Fig. 4B). A comparison of total activity between the day (with light present) and the subjective day (darkness) reveals that activity is significantly lower in the presence of light (Fig. 4B). Interestingly, subjects in the dark:dark condition appear to initially exhibit increased activity during the subjective day, before gradually reducing activity, suggesting an interaction between the effects of light and homeostatic mechanisms on activity levels. These findings are consistent with a role for light in suppressing activity, thereby inducing nocturnal behavior.

### Activity levels as related to other ecological and behavioral factors

General information regarding each species' habitat, behavior, prey preference and radiation is provided in Table 1. To determine whether any variables of rest or activity associate with these ecological factors, we compared locomotor data with known ecological variables. Species described as territorial exhibited generally higher overall activity levels compared with those characterized as weakly or non-territorial (Fig. 1C, Table 1); however, this trend did not quite reach significance in a pANOVA (*F*=2.52, *P*=0.12). We note that any conclusion about the relationship between locomotor activity and ecology may be premature, as our sampling was limited and significant differences in rest–activity behavior exist between closely related and ecologically similar species (e.g. within *Tropheops* and *Labeotropheus*). The more general conclusions to be drawn from these data is that Lake Malawi cichlids exhibit substantial and continuous variation in activity levels and patterns, and that closely related species can differ markedly in activity.

**Table 1.**
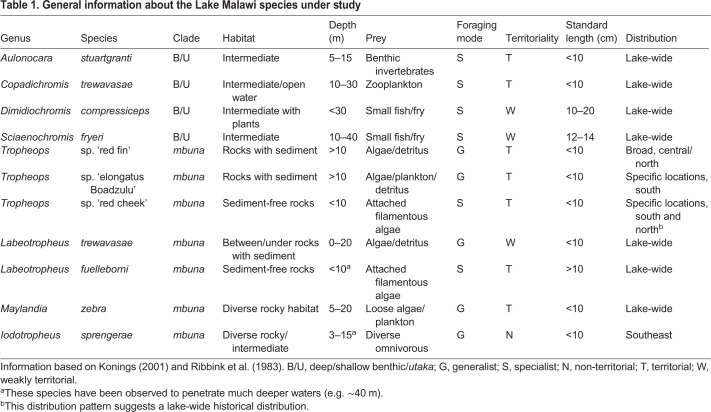
**General information about the Lake Malawi species under study**

### Eye size is associated with nighttime activity

**Fig. 5.**
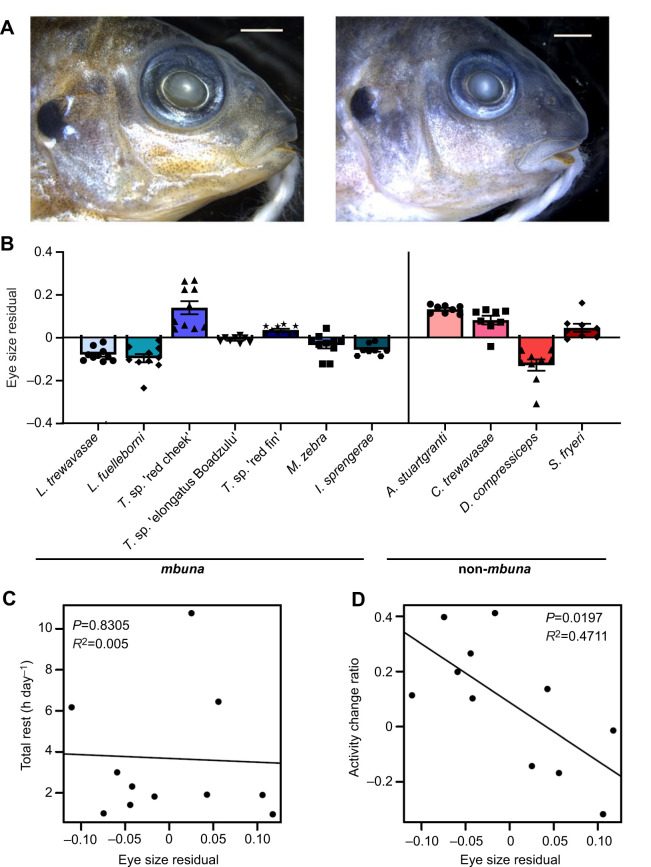
**Relationship of eye size to behavioral traits.** (A) Photographs demonstrating difference in relative eye size between *Tropheops* sp. ‘red cheek’ (left) and *Labeotropheus* *fuelleborni* (right). Both specimens were approximately 4 months old. Scale bars equal 2 mm. (B) Variation in eye size across species. (C) There is no relationship between eye size and total rest amount. (D) There is a significant negative relationship between eye size and a bias towards daytime activity. Error bars represent ±1 s.e.m. Data points represent individual animals.

Across fish species, nocturnality or adaptation to low-light conditions is associated with larger eye size (Schmitz and Motani, 2010; Schmitz and Wainwright, 2011). In addition, species that rely on visual modes of foraging generally develop larger eyes (Archer, 1999; Motani et al., 1999; Willacker et al., 2010). In contrast, species adapted to forage on attached algae generally possess smaller eyes (Hulsey et al., 2007), consistent with a functional trade-off for the production of power during jaw closure (Barel, 1982, 1983; Strauss, 1984). Specifically, algal scrapers tend to exhibit smaller and dorsally shifted eyes to accommodate larger adductor muscles that are situated below the eyes (Cooper and Westneat, 2009). To understand how eye size relates to these variables, we measured eye size in cichlid individuals in all species tested (Fig. 5A,B), and tested for significant correlations. Notably, we did not observe an obvious association between eye size and lineage or foraging mode (Fig. 5B), which is consistent with a previous report that found no correlation between eye volume and adductor muscle mass in Lake Malawi cichlids (Hulsey et al., 2007). While the visual hunting species *C. trewavasae* and *S. fryeri* possess larger eyes on average, *D. compressiceps*, an ambush hunter, has the smallest eyes of the species measured. Likewise, while the algal scraping species within the genus *Labeotropheus* has relatively small eyes, the attached algae specialist *Tropheops* sp. ‘red cheek’ has the largest relative eye size of the species measured. The other species with large eyes was *A. stuartgranti*, which is a sonar hunter with enlarged lateral line canals capable of foraging in low-light conditions (Schwalbe et al., 2012). We did not identify a correlation between rest amount and eye size (Fig. 5C). However, there was a significant correlation between eye size and preference for nighttime activity across all species (*R*^2^=0.47, *P*=0.0197; Fig. 5D), as well as within the *mbuna* (*R*^2^=0.75, *P*=0.0114; not shown). Whether the large eye size in these species represents an adaptation to nocturnality remains to be tested, but it is a notable morphological correlate worthy of further investigation.

## DISCUSSION

The diversity of the ∼3000 cichlid species throughout the world provides a unique opportunity to examine the effects of ecological niche and evolutionary history on the regulation of locomotor activity and rest. Cichlid species have undergone extensive adaptive radiations, resulting in morphologies and behaviors that can be highly specialized to specific environments. While some species exhibit high fidelity to particular habitats, many are generalists that inhabit multiple different niches. Here, we focused our analysis on Lake Malawi cichlids, a group that contains over 500 species, many of which share overlapping ecological niches (Genner et al., 2004). The well-characterized ecosystem within the lake, as well as the taxonomic diversity, uniquely positions cichlids for investigating the role of ecology in shaping behavioral evolution. Indeed, an important outstanding question is how can so many species with dietary overlap co-exist in this lake? Many factors have been proposed to contribute, including the multitude of ecological resources available in this large tropical lake, as well as the ability of cichlid species to evolve highly specific courtship and feeding behaviors (Barlow, 2008; Fryer, 1959). Circadian regulation of activity and rest may provide an additional contributor to niche partitioning, reproductive isolation and even speciation, yet these behaviors have not previously been studied systematically. The finding that the timing and duration of rest and activity varies dramatically, and continuously, between populations of Lake Malawi cichlids suggest this is a fruitful line of inquiry.

Although circadian rhythms have been studied in detail across many different animal species, surprisingly little is known about the presence and regulation of free running rhythms in teleosts. For example, Nile tilapia, *Oreochromis niloticus*, display extreme variability under light:dark conditions that ranges from diurnal to nocturnal, yet the majority of animals maintain rhythms of ∼24 h under constant dark conditions (Vera et al., 2009). Among cichlids, *O. niloticus* arguably has received the most attention regarding regulation of activity rhythms, and there is some evidence in this species for the endogenous control of circadian rhythms by melatonin, and a role for canonical clock genes (Costa et al., 2016; Martinez-Chavez et al., 2008). Feeding is likely a critical mediator of activity rhythms, though in some species, the daily timing of feeding differs from locomotor activity. For example, zebrafish are highly diurnal and maintain 24 h rhythms, yet feeding occurs primarily during the night (del Pozo et al., 2011). A similar trend has been noted in cichlids, where diurnal species exhibit mating and brooding behaviors primarily at night (Reebs, 1994; Reebs and Colgan, 1991). These findings suggest a high degree of flexibility in the circadian regulation of behavior, and that the circadian timing of many behaviors may differ from locomotor behavior that is typically used as a primary readout of the circadian clock (Reebs, 2002). Here, we focused specifically on locomotor activity and did not provide social conspecifics or food that could influence the timing of activity. Fully understanding the evolution circadian behavior of each species and its relationship to its natural environment will require examining additional behaviors that may be under circadian regulation.

A notable finding from this study is a species that appears to be nocturnal. *Tropheops* sp. ‘red cheek’ is a member of a highly speciose and ecologically diverse lineage (Albertson, 2008; Ribbink et al., 1983; Won et al., 2005). It is a vigorously territorial species that occupies the near-shore rocky habitat, where males defend large patches of rocks, cultivating algae gardens that they only allow potential mates to feed from. This species exhibits significant habitat and dietary overlap with *L. fuelleborni*, another algae-foraging species from the rocky shallows. *Labeotropheus* *fuelleborni* is arguably one of the most ecologically successful species in the lake, with numerous anatomical adaptations that enable it to dominate this niche (Albertson and Pauers, 2019; Conith et al., 2018, 2019; Konings, 2001). How then might another species coexist with such a well-adapted forager? Based on the results presented here, it is tempting to speculate that *L. fuelleborni* and *Tropheops* sp. ‘red cheek’ are partitioning their habitat by rest–activity patterns. Consistently, these two species (1) are among the most active of any measured, (2) are both strongly rhythmic and (3) their rhythmicity is opposite of one another.

Our findings raise the possibility that *Tropheops* sp. ‘red cheek’ is nocturnal in the wild, and the limited amount of night filming that has been performed in Lake Malawi supports this notion. Specifically, Arnegard and Carlson (2005) documented the nocturnal predatory behavior of the weakly electric species, *M. anguilloides*, on cichlids in the rocky habitat. The footage (available at https://malawicichlids.com/mw19000.htm) illustrates the success of the ‘pack’ hunting strategy employed by *M. anguilloides*. Two cichlid species (based on male breeding color) are readily apparent in the footage, *A. stuartgranti* and *Tropheops* sp. ‘red cheek’. Indeed, the very first fish seen in the night footage is a male *Tropheops* sp. ‘red cheek’ (at 01:15 h). This fish is not resting within a rocky cave, crack or crevice, but rather it is actively swimming well above the rocks. In fact, in the ∼6 min of night footage, no fewer than five *Tropheops* sp. ‘red cheek’ individuals can be observed, many *A. stuartgranti* are observed as well. As a point of comparison, no *Labeotropheus* or *Maylandia* species are readily observed at night, though they are common in the day footage at the beginning and end of the ∼8-min film. This filming was not intended to address questions related to rest–activity patterns in cichlids, and so we are cautious about drawing firm conclusions; however, the trends are conspicuously consistent with our laboratory results.

It is important to note that our analyses are limited to rest, and we did not examine sleep per se. Across phyla, ranging from jellyfish to humans, sleep can be defined by shared behavioral characteristics that include consolidated periods of behavioral quiescence, homeostasis following deprivation and increased arousal threshold, and species-specific posture (Keene and Duboue, 2018). In teleosts, the duration of inactivity associated with sleep has been defined as 1 min of immobility in larval *A. mexicanus* and zebrafish, and the same duration for adult *A. mexicanus* (Duboué et al., 2011; Prober et al., 2006). The duration of sleep and rest is highly variable across many other teleost species, and even between individuals of the same species. For example, different populations of *A. mexicanus* display extreme differences in sleep and activity, with cave-dwelling populations of *A. mexicanus* sleeping less than river-dwelling surface fish counterparts. These differences presumably evolved, at least in part, owing to increased foraging needs in a nutrient-poor cave environment (Aspiras et al., 2015). Based on previous work in fishes, we defined rest as the total duration of inactivity bouts longer than 1 min, and therefore these phenotypes may reflect differences in sleep duration across cichlid species. While specifically examining sleep in cichlids will require defining the period of immobility associated with changes in arousal threshold, posture and other behavioral characteristics of sleep, we submit that it represents a fruitful line of inquiry as it offers an ideal system in which to delve further into the evolution of sleep and its molecular underpinnings.

## References

[JEB242186C1] Albertson, R. C. (2008). Morphological divergence predicts habitat partitioning in a Lake Malawi cichlid species complex. *Copeia* 2008, 689-698. 10.1643/CG-07-217

[JEB242186C2] Albertson, R. C. and Pauers, M. J. (2019). Morphological disparity in ecologically diverse versus constrained lineages of Lake Malaŵi rock-dwelling cichlids. *Hydrobiologia* 832, 153-174. 10.1007/s10750-018-3829-z

[JEB242186C3] Archer, S. (ed.) (1999). *Adaptive Mechanisms in the Ecology of Vision*. Boston, MA: Kluwer Academic Publishers.

[JEB242186C4] Arnegard, M. E. and Carlson, B. A. (2005). Electric organ discharge patterns during group hunting by a mormyrid fish. *Proc. R. Soc. B Biol. Sci.* 272, 1305-1314. 10.1098/rspb.2005.3101 PMC1560340 16006329

[JEB242186C5] Aspiras, A. C., Rohner, N., Martineau, B., Borowsky, R. L. and Tabin, C. J. (2015). Melanocortin 4 receptor mutations contribute to the adaptation of cavefish to nutrient-poor conditions. *Proc. Natl. Acad. Sci. USA* 112, 9668-9673. 10.1073/pnas.1510802112 26170297 PMC4534248

[JEB242186C6] Barel, C. D. N. (1982). Towards a constructional morphology of cichlid fishes (Teleostei, Perciformes). *Netherlands J. Zool.* 33, 357-424. 10.1163/002829683X00183

[JEB242186C7] Barel, C. D. N. (1983). Form-relations in the context of constructional morphology: the eye and suspensorium of lacustrine cichlidae (Pisces, Teleostei): with a discussion on the implications for phylogenetic and allometric form-interpretations. *Netherlands J. Zool.* 34, 439-502. 10.1163/002829684X00263

[JEB242186C8] Barlow, G. (2008). *The Cichlid Fishes: Nature's Grand Experiment in Evolution*. Basic Books.

[JEB242186C9] Bayarri, M. J., Muñoz-Cueto, J. A., López-Olmeda, J. F., Vera, L. M., Rol De Lama, M. A., Madrid, J. A. and Sánchez-Vázquez, F. J. (2004). Daily locomotor activity and melatonin rhythms in Senegal sole (*Solea senegalensis*). *Physiol. Behav.* 81, 577-583. 10.1016/j.physbeh.2004.02.001 15178150

[JEB242186C72] Beale, A., Guibal, C., Tamai, T. K., Klotz, L., Cowen, S., Peyric, E., Reynoso, V. H., Yamamoto, Y. and Whitmore, D. (2013). Circadian rhythms in Mexican blind cavefish *Astyanax mexicanus* in the lab and in the field. *Nat. Commun.* **4,** 2769. 10.1038/ncomms3769 24225650

[JEB242186C10] Brawand, D., Wagner, C. E., Li, Y. I., Malinsky, M., Keller, I., Fan, S., Simakov, O., Ng, A. Y., Lim, Z. W., Bezault, E. et al. (2014). The genomic substrate for adaptive radiation in African cichlid fish. *Nature* 513, 375-381. 10.1038/nature13726 25186727 PMC4353498

[JEB242186C11] Brown, E. B., Torres, J., Bennick, R. A., Rozzo, V., Kerbs, A., DiAngelo, J. R., Keene, A. C. (2018). Variation in sleep and metabolic function is associated with latitude and average temperature in *Drosophila melanogaster*. *Ecol. Evol.* 8, 4084-4097. 10.1002/ece3.3963 29721282 PMC5916307

[JEB242186C71] Cavallari, N., Frigato, E., Vallone, D., Fröhlich, N., Lopez-Olmeda, J. F., Foà, A., Berti, R., Sánchez-Vázquez, F. J., Bertolucci, C. and Foulkes, N. S. (2011). A blind circadian clock in cavefish reveals that opsins mediate peripheral clock photoreception. *PLoS Biol.* **9**, e1001142. 10.1371/journal.pbio.1001142 PMC3167789 21909239

[JEB242186C12] Conith, M. R., Hu, Y., Conith, A. J., Maginnis, M. A., Webb, J. F. and Craig Albertson, R. (2018). Genetic and developmental origins of a unique foraging adaptation in a Lake Malawi cichlid genus. *Proc. Natl. Acad. Sci. USA* 115, 7063-7068. 10.1073/pnas.1719798115 29915062 PMC6142203

[JEB242186C13] Conith, M. R., Conith, A. J. and Albertson, R. C. (2019). Evolution of a soft-tissue foraging adaptation in African cichlids: roles for novelty, convergence, and constraint. *Evolution* 73, 2072-2084. 10.1111/evo.13824 31418824

[JEB242186C14] Conith, A. J., Kidd, M. R., Kocher, T. D. and Albertson, R. C. (2020a). Ecomorphological divergence and habitat lability in the context of robust patterns of modularity in the cichlid feeding apparatus. *BMC Evol. Biol.* 20, 95. 10.1186/s12862-020-01648-x 32736512 PMC7393717

[JEB242186C15] Conith, A. J., Hope, S. A., Chhouk, B. H. and Craig Albertson, R. (2020b). Weak genetic signal for phenotypic integration implicates developmental processes as major regulators of trait covariation. *Mol. Ecol.* 30, 464-480. 10.1111/mec.15748 33231336 PMC8811731

[JEB242186C16] Cooper, W. J. and Westneat, M. W. (2009). Form and function of damselfish skulls: rapid and repeated evolution into a limited number of trophic niches. *BMC Evol. Biol.* 9, 24. 10.1186/1471-2148-9-24 19183467 PMC2654721

[JEB242186C17] Costa, L. S., Serrano, I., Sánchez-Vázquez, F. J. and López-Olmeda, J. F. (2016). Circadian rhythms of clock gene expression in Nile tilapia (*Oreochromis niloticus*) central and peripheral tissues: influence of different lighting and feeding conditions. *J. Comp. Physiol. B Biochem. Syst. Environ. Physiol.* 186, 775-785. 10.1007/s00360-016-0989-x 27085855

[JEB242186C18] del Pozo, A., Sánchez-Férez, J. A. and Sánchez-Vázquez, F. J. (2011). Circadian rhythms of self-feeding and locomotor activity in zebrafish (*Danio rerio*). *Chronobiol. Int.* 28, 39-47. 10.3109/07420528.2010.530728 21182403

[JEB242186C19] Duboué, E. R., Keene, A. C. and Borowsky, R. L. (2011). Evolutionary convergence on sleep loss in cavefish populations. *Curr. Biol.* 21, 671-676. 10.1016/j.cub.2011.03.020 21474315

[JEB242186C20] Edgley, D. E. and Genner, M. J. (2019). Adaptive diversification of the lateral line system during cichlid fish radiation. *iScience* 16, 1-11. 10.1016/j.isci.2019.05.016 31146127 PMC6542376

[JEB242186C21] Feng, N. Y. and Bass, A. H. (2016). ‘Singing’ fish rely on circadian rhythm and melatonin for the timing of nocturnal courtship vocalization. *Curr. Biol.* 26, 2681-2689. 10.1016/j.cub.2016.07.079 27666972

[JEB242186C22] Freckleton, R. P., Harvey, P. H. and Pagel, M. (2002). Phylogenetic analysis and comparative data: a test and review of evidence. *Am. Nat.* 160, 712-726. 10.1086/343873 18707460

[JEB242186C23] Fryer, G. (1959). Some aspects of evolution in Lake Nyasa. *Evolution* 13, 440-451. 10.1111/j.1558-5646.1959.tb03034.x

[JEB242186C24] Gandhi, A. V., Mosser, E. A., Oikonomou, G. and Prober, D. A. (2015). Melatonin Is required for the circadian regulation of sleep. *Neuron* 85, 1193-1199. 10.1016/j.neuron.2015.02.016 25754820 PMC4851458

[JEB242186C25] Genner, M. J., Seehausen, O., Cleary, D. F. R., Knight, M. E., Michel, E. and Turner, G. F. (2004). How does the taxonomic status of allopatric populations influence species richness within African cichlid fish assemblages? *J. Biogeogr.* 31, 93-102. 10.1046/j.0305-0270.2003.00986.x

[JEB242186C26] Hammond, T. T., Palme, R. and Lacey, E. A. (2018). Ecological specialization, variability in activity patterns and response to environmental change. *Biol. Lett.* 14, 20180115. 10.1098/rsbl.2018.0115 29950317 PMC6030591

[JEB242186C27] Harmon, L. J., Weir, J. T., Brock, C. D., Glor, R. E. and Challenger, W. (2008). GEIGER: investigating evolutionary radiations. *Bioinformatics* 24, 129-131. 10.1093/bioinformatics/btm538 18006550

[JEB242186C28] Hoogenboom, I., Daan, S., Dallinga, J. H. and Schoenmakers, M. (1984). Seasonal change in the daily timing of behaviour of the common vole, *Microtus arvalis*. *Oecologia* 61, 18-31. 10.1007/BF00379084 28311381

[JEB242186C29] Howland, H. C., Merola, S. and Basarab, J. R. (2004). The allometry and scaling of the size of vertebrate eyes. *Vision Res.* 44, 2043-2065. 10.1016/j.visres.2004.03.023 15149837

[JEB242186C30] Huang, W., Ramsey, K. M., Marcheva, B. and Bass, J. (2011). Circadian rhythms, sleep, and metabolism. *J. Clin. Invest.* 121, 2133-2141. 10.1172/JCI46043 21633182 PMC3104765

[JEB242186C31] Huber, R., Van staaden, M. J., Kaufman, L. S. and Liem, K. F. (1997). Microhabitat use, trophic patterns, and the evolution of brain structure in African cichlids. *Brain. Behav. Evol.* 50, 167-182. 10.1159/000113330 9288416

[JEB242186C32] Hulsey, C. D., Mims, M. C. and Streelman, J. T. (2007). Do constructional constraints influence cichlid craniofacial diversification? *Proc. R. Soc. B Biol. Sci.* 274, 1867-1875. 10.1098/rspb.2007.0444 PMC2270932 17519189

[JEB242186C33] Iigo, M. and Tabata, M. (1996). Circadian rhythms of locomotor activity in the goldfish *Carassius auratus*. *Physiol. Behav.* 60, 775-781. 10.1016/0031-9384(96)00131-X 8873250

[JEB242186C34] Jaggard, J. B., Lloyd, E., Lopatto, A., Duboue, E. R. and Keene, A. C. (2019). Automated measurements of sleep and locomotor activity in Mexican cavefish. *J. Vis. Exp.* 145, e59198. 10.3791/59198 30958465

[JEB242186C35] Karvonen, A., Wagner, C. E., Selz, O. M. and Seehausen, O. (2018). Divergent parasite infections in sympatric cichlid species in Lake Victoria. *J. Evol. Biol.* 31, 1313-1329. 10.1111/jeb.13304 29944770

[JEB242186C36] Keene, A. C. and Duboue, E. R. (2018). The origins and evolution of sleep. *J. Exp. Biol.* 221, jeb159533 10.1242/jeb.159533 PMC6515771 29895581

[JEB242186C37] Konings, A. F. (2001). *Malawi Cichlids in their Natural Habitat*, 3rd edn. Cichlid Press.

[JEB242186C38] Malinsky, M., Challis, R. J., Tyers, A. M., Schiffels, S., Terai, Y., Ngatunga, B. P., Miska, E. A., Durbin, R., Genner, M. J. and Turner, G. F. (2015). Genomic islands of speciation separate cichlid ecomorphs in an East African crater lake. *Science* 350, 1493-1498. 10.1126/science.aac9927 26680190 PMC4700518

[JEB242186C39] Malinsky, M., Svardal, H., Tyers, A. M., Miska, E. A., Genner, M. J., Turner, G. F. and Durbin, R. (2018). Whole-genome sequences of Malawi cichlids reveal multiple radiations interconnected by gene flow. *Nat. Ecol. Evol.* 2, 1940-1955. 10.1038/s41559-018-0717-x 30455444 PMC6443041

[JEB242186C40] Martinez-Chavez, C. C., Al-Khamees, S., Campos-Mendoza, A., Penman, D. J. and Migaud, H. (2008). Clock-controlled endogenous melatonin rhythms in Nile tilapia (*Oreochromis niloticus niloticus*) and African catfish (*Clarias gariepinus*). *Chronobiol. Int.* 25, 31-49. 10.1080/07420520801917547 18293148

[JEB242186C41] Maruyama, A., Rusuwa, B. and Yuma, M. (2010). Asymmetric interspecific territorial competition over food resources amongst Lake Malawi cichlid fishes. *African Zool.* 45, 24-31. 10.1080/15627020.2010.11657251

[JEB242186C42] McGee, M. D., Borstein, S. R., Meier, J. I., Marques, D. A., Mwaiko, S., Taabu, A., Kishe, M. A., O'Meara, B., Bruggmann, R., Excoffier, L. et al. (2020). The ecological and genomic basis of explosive adaptive radiation. *Nature* 586, 75-79. 10.1038/s41586-020-2652-7 32848251

[JEB242186C43] Motani, R., Rothschild, B. M. and Wahl, W. (1999). Large eyeballs in diving ichthyosaurs. *Nature* 402, 747. 10.1038/45435 10927017

[JEB242186C44] Oliveira, C., Garcia, E. M., López-Olmeda, J. F. and Sánchez-Vázquez, F. J. (2009). Daily and circadian melatonin release in vitro by the pineal organ of two nocturnal teleost species: Senegal sole (*Solea senegalensis*) and tench (*Tinca tinca*). *Comp. Biochem. Physiol. A Mol. Integr. Physiol.* 153, 297-302. 10.1016/j.cbpa.2009.03.001 19272458

[JEB242186C45] Pagel, M. (1999). Inferring the historical patterns of biological evolution. *Nature* 401, 877-884. 10.1038/44766 10553904

[JEB242186C46] Parnell, N. F. and Todd Streelman, J. (2011). The macroecology of rapid evolutionary radiation. *Proc. R. Soc. B Biol. Sci.* 278, 2486-2494. 10.1098/rspb.2010.1950 PMC3125616 21208961

[JEB242186C47] Prober, D. A., Rihel, J., Onah, A. A., Sung, R. J. and Schier, A. F. (2006). Hypocretin/orexin overexpression induces an insomnia-like phenotype in zebrafish. *J. Neurosci.* 26, 13400-13410. 10.1523/JNEUROSCI.4332-06.2006 17182791 PMC6675014

[JEB242186C48] Raphael, K. A., Sved, J. A., Pearce, S., Oakeshott, J. G., Gilchrist, A. S., Sherwin, W. B. and Frommer, M. (2019). Differences in gene regulation in a tephritid model of prezygotic reproductive isolation. *Insect Mol. Biol.* 28, 689-702. 10.1111/imb.12583 30955213

[JEB242186C49] Reebs, S. G. (1994). Nocturnal mate recognition and nest guarding by female convict cichlids (Pisces, Cichlidae: *Cichlasoma mgrofasciatum*). *Wiley Online Libr.* 96, 303-312. 10.1111/j.1439-0310.1994.tb01018.x

[JEB242186C50] Reebs, S. G. (2002). Plasticity of diel and circadian activity rhythms in fishes. *Rev. Fish Biol. Fish.* 12, 349-371. 10.1023/A:1025371804611

[JEB242186C51] Reebs, S. G. and Colgan, P. W. (1991). Nocturnal care of eggs and circadian rhythms of fanning activity in two normally diurnal cichlid fishes, *Cichlasoma nigrofasciatum* and *Herotilapia multispinosa*. *Anim. Behav.* 41, 303-311. 10.1016/S0003-3472(05)80482-8

[JEB242186C52] Reebs, S. G. and Colgan, P. W. (1992). Proximal cues for nocturnal egg care in convict cichlids, *Cichlasoma nigrofasciatum*. *Anim. Behav.* 43, 209-214. 10.1016/S0003-3472(05)80216-7

[JEB242186C53] Ribbink, A. J., Marsh, B. A., Marsh, A. C., Ribbink, A. C. and Sharp, B. J. (1983). A preliminary survey of the cichlid fishes of rocky habitats in Lake Malawi. *South African J. Zool.* 18, 149-310. 10.1080/02541858.1983.11447831

[JEB242186C54] Schmitz, L. and Motani, R. (2010). Morphological differences between the eyeballs of nocturnal and diurnal amniotes revisited from optical perspectives of visual environments. *Vision Res.* 50, 936-946. 10.1016/j.visres.2010.03.009 20304001

[JEB242186C55] Schmitz, L. and Wainwright, P. C. (2011). Nocturnality constrains morphological and functional diversity in the eyes of reef fishes. *BMC Evol. Biol.* 11, 338. 10.1186/1471-2148-11-338 22098687 PMC3240680

[JEB242186C56] Schneider, C. A., Rasband, W. S. and Eliceiri, K. W. (2012). NIH Image to ImageJ: 25 years of image analysis. *Nat. Methods* 9, 671-675. 10.1038/nmeth.2089 22930834 PMC5554542

[JEB242186C57] Schwalbe, M. A. B., Bassett, D. K. and Webb, J. F. (2012). Feeding in the dark: Lateral-line-mediated prey detection in the peacock cichlid *Aulonocara stuartgranti*. *J. Exp. Biol.* 215, 2060-2071. 10.1242/jeb.065920 22623194

[JEB242186C58] Siegel, J. M. (2005). Clues to the functions of mammalian sleep. *Nature* 437, 1264-1271. 10.1038/nature04285 16251951 PMC8760626

[JEB242186C59] Siegel, J. M. (2009). Sleep viewed as a state of adaptive inactivity. *Nat. Rev. Neurosci.* 10, 747-753. 10.1038/nrn2697 19654581 PMC8740608

[JEB242186C60] Smith, P. H. (1979). Genetic manipulation of the circadian clock's timing of sexual behaviour in the Queensland fruit flies, *Dacus tryoni* and *Dacus neohumeralis*. *Physiol. Entomol.* 4, 71-78. 10.1111/j.1365-3032.1979.tb00179.x

[JEB242186C61] Strauss, R. E. (1984). Allometry and functional feeding morphology in haplochromine cichlids. In *Evolution of Fish Species Flocks*, (ed. A. A. Echelle and I. Kornfield) pp. 217-229.

[JEB242186C62] Terai, Y., Miyagi, R., Aibara, M., Mizoiri, S., Imai, H., Okitsu, T., Wada, A., Takahashi-Kariyazono, S., Sato, A., Tichy, H. et al. (2017). Visual adaptation in Lake Victoria cichlid fishes: depth-related variation of color and scotopic opsins in species from sand/mud bottoms. *BMC Evol. Biol.* 17, 200. 10.1186/s12862-017-1040-x PMC5568302 28830359

[JEB242186C63] Tung Ho, L. si and Ané, C. (2014). A linear-time algorithm for Gaussian and non-Gaussian trait evolution models. *Syst. Biol.* 63, 397-408. 10.1093/sysbio/syu005 24500037

[JEB242186C64] Turner, G. F., Seehausen, O., Knight, M. E., Allender, C. J. and Robinson, R. L. (2001). How many species of cichlid fishes are there in African lakes? *Mol. Ecol.* 10, 793-806. 10.1046/j.1365-294x.2001.01200.x 11298988

[JEB242186C65] Vaze, K. M. and Sharma, V. K. (2013). On the adaptive significance of circadian clocks for their owners. *Chronobiol. Int.* 30, 413-433. 10.3109/07420528.2012.754457 23452153

[JEB242186C66] Vera, L. M., Cairns, L., Sánchez-Vázquez, F. J. and Migaud, H. (2009). Circadian rhythms of locomotor activity in the Nile tilapia *Oreochromis niloticus*. *Chronobiol. Int.* 26, 666-681. 10.1080/07420520902926017 19444748

[JEB242186C67] Willacker, J. J., Von Hippel, F. A., Wilton, P. R. and Walton, K. M. (2010). Classification of threespine stickleback along the benthic–limnetic axis. *Biol. J. Linn. Soc.* 101, 595-608. 10.1111/j.1095-8312.2010.01531.x PMC3017379 21221422

[JEB242186C68] Won, Y. J., Sivasundar, A., Wang, Y. and Hey, J. (2005). On the origin of Lake Malawi cichlid species: a population genetic analysis of divergence. *Proc. Natl. Acad. Sci. USA* 102, 6581-6586. 10.1073/pnas.0502127102 15851665 PMC1131877

[JEB242186C69] Yoshizawa, M., Robinson, B. G., Duboué, E. R., Masek, P., Jaggard, J. B., O'Quin, K. E., Borowsky, R. L., Jeffery, W. R. and Keene, A. C. (2015). Distinct genetic architecture underlies the emergence of sleep loss and prey-seeking behavior in the Mexican cavefish. *BMC Biol.* 13, 15. 10.1186/s12915-015-0119-3 25761998 PMC4364459

[JEB242186C70] Zhdanova, I. V., Wang, S. Y., Leclair, O. U. and Danilova, N. P. (2001). Melatonin promotes sleep-like state in zebrafish. *Brain Res.* 903, 263-268. 10.1016/S0006-8993(01)02444-1 11382414

